# Subjective caregiver burden and anxiety in informal caregivers: A systematic review and meta-analysis

**DOI:** 10.1371/journal.pone.0247143

**Published:** 2021-03-01

**Authors:** Rafael del-Pino-Casado, Emilia Priego-Cubero, Catalina López-Martínez, Vasiliki Orgeta

**Affiliations:** 1 Faculty of Health Sciences, Department of Nursing, University of Jaén, Jaén, Andalusia, Spain; 2 Division of Psychiatry, University College London, London, United Kingdom; University of Bologna, ITALY

## Abstract

There is increasing evidence that subjective caregiver burden is an important determinant of clinically significant anxiety in family carers. This meta-analysis aims to synthesise this evidence and investigate the relationship between subjective caregiver burden and anxiety symptoms in informal caregivers. We searched PubMed, CINAHL and PsycINFO up to January 2020. Combined estimates were obtained using a random-effects model. After screening of 4,312 articles, 74 studies (with 75 independent samples) were included. There was a large, positive association between subjective caregiver burden and anxiety symptoms (r = 0.51; 95% CI = 0.47, 0.54; I^2^ = 0.0%). No differences were found in subgroup analyses by type of study design (cross-sectional vs. longitudinal), sampling, control of confounders or care-recipient characteristics. Subjective caregiver burden is an important risk factor for anxiety in informal caregivers. Targeting subjective caregiver burden could be beneficial in preventing clinically significant anxiety for the increasing number of family carers worldwide.

## Introduction

Research has consistently shown that informal family caregiving is often a burdensome role with negative consequences on carers’ physical and emotional health [1,2]. In contrast to formal caregivers, informal carers typically provide some form of unpaid, ongoing assistance with activities of daily living (ADLs) or instrumental activities of daily living (IADLs) to a person with a chronic illness or disability [3]. Due to increases in life expectancy and associated dependency, family carers will continue to be the main source of care for many people in both developed and developing countries [4]. As a result, caregiver burden will continue to be a significant public healthcare issue for many years to come affecting the quality of life of millions of people world-wide [5–7].

The negative health consequences of caregiving take place over many years affecting both the physical and emotional health of carers. Several studies have consistently reported that informal carers have an increased risk of experiencing psychiatric disorders [1,7,8]. Although anxiety disorders appear to be the most prevalent, they remain the least studied in the context of informal caregiving [1,7,8]. Anxiety is a highly distressing condition that is of particular importance in the context of caregiving [8]. Anxiety symptoms affect the daily life of informal carers [1,7,8], and accompanied symptoms such as feelings of worry and fear [9], can interfere with caregiving duties and negatively impact both carer and care recipient outcomes [1]. Identifying therefore which factors predispose carers to increasing levels of anxiety is essential for early detection and prevention of these symptoms.

Both theoretical [10] and empirical work [11–13] suggests that subjective caregiver burden is the most important correlate of carer anxiety. Despite several definitions in the literature, subjective caregiver burden is best conceptualised as a multidimensional concept that incorporates emotional, physical, social and economic aspects of the caregiving role, considered to be unique for each caregiver [14]. Several studies have examined the association between subjective caregiver burden and anxiety symptoms across several caregiving groups including carers of dependent older people [15], carers of people living with dementia [16], and those caring for cancer survivors [17]. Despite several reviews on the topic [18–20] there is currently no effect estimate on the association between subjective caregiver burden and anxiety symptoms in carers, and no systematic reviews combining evidence across all caregiving groups. As a result, the quality of evidence to date remains unknown and there are no reviews assessing the influence of potential moderators on the relationship between subjective caregiver burden and anxiety. Identifying moderators of this relationship is important for understanding mechanisms linking subjective caregiver burden and anxiety symptoms.

In this study our aim was to provide the first systematic review on the meta-analytic relationship between anxiety symptoms and subjective caregiver burden. We considered this analysis as very important in providing a quantitative estimate of the strength of the population effect size and a much-needed synthesis in the area. Therefore, the aim of this study was to conduct the first systematic review in the literature on the relationship between subjective caregiver burden and anxiety symptoms across all caregiving groups, determine the strength of the association and investigate the role of potential moderators to inform future research in the area.

## Material and methods

### Design

The present study is a systematic review and meta-analysis of original quantitative studies reporting on the relationship between subjective caregiver burden and anxiety symptoms across caregiving populations, in line with the recommendations of the COSMOS-E guide [21].

### Search strategy

Unlimited time searches were performed using the following search terms: anxiety and burden (or strain or role overload) and caregivers (or caregiv* or carer*). We searched PubMed, CINAHL (EBSCO) and PsycINFO (ProQuest) up to 31 of January of 2020. An open search, without filters, was performed to maximize sensitivity. We performed additional hand searches of relevant reviews in the area and contacted authors for new studies. We requested data that did not appear in the original articles.

### Eligibility criteria

#### Types of studies

Inclusion criteria of studies were: a) original studies on informal carers of dependent adults, carrying out caregiving duties at the home of the person they cared for, b) assessing subjective caregiver burden and anxiety symptoms using a valid tool, and d) those providing data to calculate an appropriate effect size. Both cross-sectional and longitudinal studies were included. Study selection was made by two reviewers independently and disagreements were resolved by consensus with a third reviewer. Studies that did not report a correlation coefficient or another statistical metric that allowed calculation of a correlation coefficient were excluded.

#### Types of participants

Informal carers: Carers of dependent people, defined as those individuals who provided unpaid care to a "dependent person” or “care recipient”. Care recipients: individuals requiring support with at least one basic or instrumental activity of daily living.

### Methodological quality criteria

Following the recommendations of Boyle [22] and Viswanathan et al. [23], we used the following criteria for assessing the methodological quality of individual studies: (1) sampling (use of probabilistic sampling or not), (2) reliability and validity of measures (content validity and internal consistency of measures in the target population or similar); (3) control for confounding factors (controlling for at least one measure of objective caregiver burden) and (4) for longitudinal studies: absence of attrition (≥ 80% follow-up rate of the original population taking part in the study). Criterion 2 was mandatory for a study to be included in the meta-analysis. Two reviewers (RdPC and EPC) assessed quality of studies independently and any disagreements were resolved by consensus with a third reviewer.

Controlling for objective caregiver burden was considered a necessary quality criterion for studies due to its association with subjective caregiver burden [24] and anxiety symptoms [25]. Measures of objective burden included in studies were functional capacity, cognitive impairment, behavioural problems experienced by the care recipient, and intensity of care provided [26]. Given that measures of objective burden are highly intercorrelated [27], we considered as high quality any study that controlled for at least one measure of objective burden in the design and/or analysis (e.g. through multivariate analysis) via allocation between groups (e.g., through stratification or matching) or studies controlling for confounding variables in the design and/or analysis (e.g. through multivariate analysis) [23]. If statistical adjustment was performed, we considered no confounding bias to be present if variation of the point estimate was less than 10% [28].

Based on the Grading of Recommendations Assessment, Development and Evaluation (GRADE) [29], we additionally evaluated imprecision, inconsistency and risk of publication bias. Imprecision was evaluated in line with guidelines [30], which comprise: a) number of included studies in a meta-analysis (large: >10 studies, moderate: 5–10 studies and small: <5 studies) and b) median sample size (high: >300 participants, intermediate: 100–300 and low: <100). Inconsistency was measured through heterogeneity of findings in individual studies. Publication bias was assessed by a funnel plot and statistical tests.

### Data extraction

Two reviewers (RdPC and EPC) extracted data independently using a standardized data extraction form, which included information on type of study design, sample size, health condition/illness of care recipients, quality criteria and effect sizes. Disagreements were resolved by consensus.

### Synthesis of information

The estimated effect measure used was the correlation coefficient. Following the recommendations of Cooper et al. [31], a random effects model was used to calculate the combined effect, in order to generalize findings across caregiving groups. For longitudinal studies with repeated measures reporting cross-sectional correlations in each time point, the first correlation was selected. We measured heterogeneity with the Cochran Q [32], considering p values above 0.10 indicative of no heterogeneity. We used the I^2^ (Higgins et al. [33]) to measure the proportion of heterogeneity due to the variability of effect estimates amongst individual studies, with values of 25, 50 and 75% indicative of mild, moderate and severe heterogeneity, respectively.

We assessed publication bias through evaluating asymmetry in a funnel plot, a p value above 0.10 on the Egger test [34] and the Trim and Fill method which computes the combined effect in the context of absence of publication bias [35]. We performed sensitivity analysis removing one study each time to evaluate the robustness of the results of the meta-analysis (leave-one-out method), and several subgroup analyses to investigate differences of the combined effect due to study design, methodological quality of individual studies and health condition of care recipients (i.e. frailty, dementia, cancer, stroke). We performed all analyses using Comprehensive Meta-analysis 3.3.

## Results

### Study characteristics

**Fig 1:**
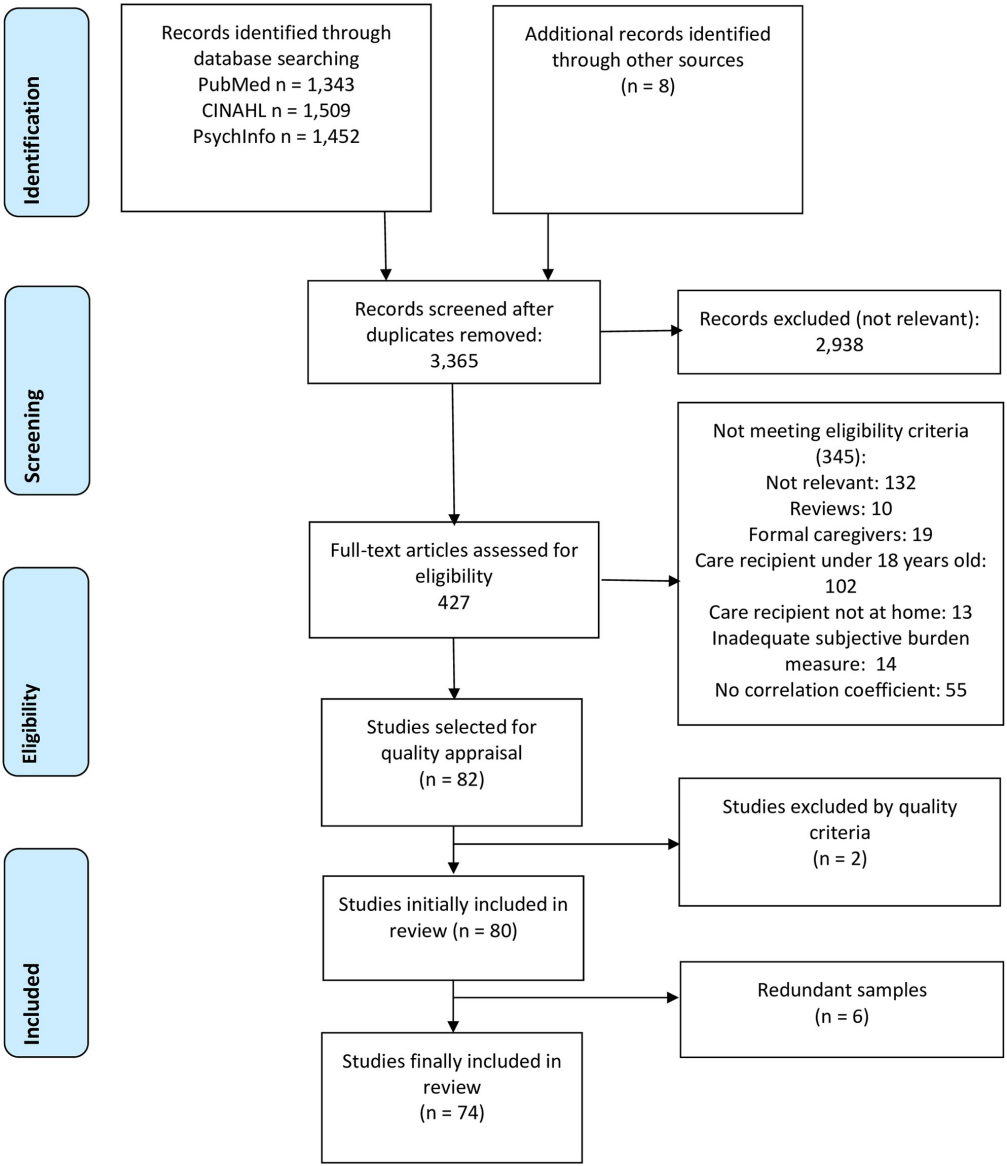
Flow diagram (PRISMA).

A total of 4,304 references were retrieved from the search with a further eight additional articles identified via other sources. After removing duplicates, 3,365 records were screened, of which 2,938 were excluded as not relevant, leaving 427 articles to be assessed for eligibility. Of these 345 were excluded as not relevant or not meeting inclusion criteria, leaving a total of 82 full text articles assessed for quality appraisal (see Fig 1). Of these 82 studies, two studies were excluded as not meeting mandatory criteria for methodological quality [36,37] and six studies [38–43] reported secondary analyses of a primary study (samples already included). Finally, 74 studies with 75 independent samples, met inclusion criteria and were included in the review [11–17,44–110].

Characteristics of the included studies are presented in Table 1. Care recipients included people living with a diagnosis of dementia (24 studies), frail older people (11 studies with 12 samples), people living with cancer (12 studies) and stroke survivors (eight studies). All but nine studies employed a cross-sectional design. Those utilising a longitudinal design used repeated measures, with one study providing only cross-sectional correlations in each time point. Therefore, a total of 66 studies with 67 independent samples provided cross-sectional correlations and eight studies with eight independent samples contributed longitudinal correlations.

**Table 1:** Description of the studies included in the review (I).

Studies (author, year)	N	Design	Care recipients	Measure of burden	Measure of anxiety
Adejumo et al., 2019 [44]	57	Cross-sectional	Chronic kidney failure	ZBI	HADS
Alfaro-Ramirez del Castillo et al., 2008 [45]	100	Cross-sectional	Cancer	ZBI	MLSI
Ar, 2017 [46]	190	Cross-sectional	Dementia	ZBI	STAI-S
Bozkurt Zincir et al., 2014 [47]	138	Cross-sectional	Other chronic conditions	ZBI	STAI-S
Buscemi et al., 2010 [14]	59	Cross-sectional	Cancer	BCOS	HADS
Carod-Artal et al., 2009 [48]	200	Cross-sectional	Stroke	ZBI	HADS
Chan et al., 2018 [49]	274	Cross-sectional	Frail older people	ZBI	HADS
Chattat et al., 2011 [50]	273	Cross-sectional	Dementia	ZBI	HADS
Coleman et al., 2015 [51]	40	Cross-sectional	Spinal cord injury	ZBI	STAI
Cooper et al. 2008 [52]	93	Repeated measures	Dementia	ZBI	HADS
del-Pino-Casado et al., 2015 [53]	200	Cross-sectional	Frail older people	CSI	Goldberg
del-Pino-Casado et al., 2019 [54]	178	Repeated measures	Frail older people	CSI	Goldberg
Dikeç et al., 2018 [55]	481	Cross-sectional	Mental Illness	PFBS	BAI
Dos Santos et al., 2017 [56]	36	Cross-sectional	Mental Illness	ZBI	BAI
Edelstein et al., 2018 [15]	107	Cross-sectional	Frail older people	ZBI	HADS
Efi et al., 2017 [57]	150	Cross-sectional	Stroke	BCOS	HADS
Fu et al., 2007 [58]	42	Cross-sectional	Dementia	ZBI	SAS
Galindo Vázquez et al., 2015 [59]	200	Cross-sectional	Cancer	ZBI	HADS
Garand et al., 2005 [60]	27	Cross-sectional	Dementia	SBS	STAI-S
Garcia-Alberca et al., 2012 [11]	80	Cross-sectional	Dementia	ZBI	STAI-S
Goetzinger et al., 2012 [61]	610	Cross-sectional	Other chronic conditions	SCB	STAI-S
Gonzalez-Abraldes et al., 2013 [62]	33	Cross-sectional	Dementia	ZBI	STAI-T
Govina et al., 2015 [63]	100	Cross-sectional	Cancer	BCOS	HADS
Guedes and Pereira, 2013 [64]	50	Cross-sectional	Frail older people	ZBI	DASS
Hu et al., 2018 [13]	117	Cross-sectional	Stroke	ZBI	HARS
Iavarone et al., 2014 [65]	86	Cross-sectional	Dementia	CBI	STAI-T
Jaracz et al., 2014 [66]	150	Cross-sectional	Frail older people	CBS 1	HADS
Jones et al., 2015 [67]	76	Cross-sectional	Cancer	BASC	DASS
Karabekiroglu et al., 2018 [68]	60	Cross-sectional	Cancer	ZBI	BAI
Kemp et al., 2018 [69]	44	Cross-sectional	Cancer	OCBS	HADS
Kruithof et al., 2016 [12]	183	Repeated measures	Stroke	CSI	HADS
Lee et al., 2010 [70]	81	Cross-sectional	Other chronic conditions	CBS 2	HADS
Leibach et al., 2014 [71]	90	Cross-sectional	Traumatic brain injury	ZBI	STAI
Liu et al., 2012 Liu et al. [72]	180	Cross-sectional	Frail older people	CBI	SAS
López Alonso and Moral Serrano, 2005 [73]	215	Cross-sectional	Other chronic conditions	CSI	Goldberg
López-Martínez, 2019 [74]	81	Repeated measures	Frail older people	CSI	Goldberg
Macías-Delgado Yanet et al., 2014 [75]	35	Cross-sectional	Multiple sclerosis	ZBI	ISRA
Majestic and Eddington, 2019 [76]	102	Cross-sectional	Cancer	ZBI	DASS
Manso Martínez et al., 2013 (men) [77]	14	Cross-sectional	Frail older people	ZBI	HADS
Manso Martínez et al., 2013 (women) [77]	74	Cross-sectional	Frail older people	ZBI	HADS
Mavardi et al., 2005 [78]	419	Cross-sectional	Dementia	CBI	BSI
McCullagh et al., 2005 [79]	232	Repeated measures	Stroke	CBS	HADS
Medrano et al., 2014 [80]	67	Cross-sectional	Dementia	ZBI	HARS
Mei et al., 2018 [81]	145	Cross-sectional	Stroke	CBI	HADS
Méndez et al., 2010 [82]	14	Cross-sectional	Dementia	ZBI	STAI
Molina Linde and Iañez Velasco 2006 [83]	46	Cross-sectional	Dementia	ZBI	STAI-s
Morlett Paredes, 2014 [84]	102	Cross-sectional	Mental Illness	ZBI	GAD
Özyesil et al., 2014 [85]	140	Cross-sectional	Frail older people	CBI	STAI-S
Pagnini et al., 2010 [86]	40	Cross-sectional	Multiple sclerosis	ZBI	STAI
Palacio et al., 2018 [87]	50	Cross-sectional	Cancer	ZBI	HADS
Perez Cruz et al., 2019 [88]	198	Cross-sectional	Frail older people	CSI	HARS
Pérez-Ordóñez et al., 2016 [17]	50	Cross-sectional	Cancer	CSI	Goldberg
Perpiña-Galvañ et al., 2019 [89]	78	Cross-sectional	Cancer	ZBI	HADS
Raveis et al., 2000 [90]	164	Cross-sectional	Cancer	CRA	STAI-S
Razani et al., 2014 [91]	44	Repeated measures	Dementia	CBI	BSI
Romero-Moreno et al., 2011 [92]	167	Cross-sectional	Dementia	ZBI	POMS
Sadak et al., 2017 [93]	227	Cross-sectional	Dementia	KCSS	GAD
Sanyal et al., 2015 [94]	150	Cross-sectional	Parkinson	ZBI	HADS
Shukri et al., 2020 [95]	340	Cross-sectional	Chronic kidney failure	BSFC	HADS
Stanley et al., 2017 [96]	75	Cross-sectional	Mental Illness	ZBI	DASS
Stevens et al., 2013 [111]	90	Cross-sectional	Traumatic brain injury	ZBI	STAI-S
Tang et al., 2011 [98]	123	Cross-sectional	Stroke	CBS 1	HADS
Torny et al., 2018 [99]	38	Cross-sectional	Parkinson	ZBI	HADS
Trapp et al., 2015 [100]	40	Cross-sectional	Spinal cord injury	ZBI	STAI
Tremont et al., 2006 [101]	72	Cross-sectional	Dementia	ZBI	STAI
Truzzi et al., 2008 [102]	69	Cross-sectional	Dementia	ZBI	BAI
Tsatali et al., 2019 [103]	247	Cross-sectional	Dementia	ZBI	BAI
Vérez Cotelo et al., 2015 [104]	25	Cross-sectional	Dementia	ZBI	STAI-S
Vitaliano et al., 1991 [105]	79	Repeated measures	Dementia	SCB	SCL
Wang et al., 2008 [106]	42	Cross-sectional	Dementia	ZBI	HARS
Wang et al., 2018 [16]	210	Cross-sectional	Dementia	CBI	SAS
Winslow, 1997 [107]	452	Repeated measures	Dementia	Study specific	HSCL
Yu et al., 2018 [108]	327	Cross-sectional	Mental Illness	ZBI	GAD
Zawadzki et al., 2011 [109]	51	Cross-sectional	Dementia	ZBI	Study specific
Zhu and Jiang, 2018 [110]	202	Repeated measures (*)	Stroke	BCOS	HARS

(*) With cross-sectional correlations.

Note: Abbreviations of measures are presented in S1 Appendix.

### Anxiety symptoms and subjective caregiver burden

**Fig 2:**
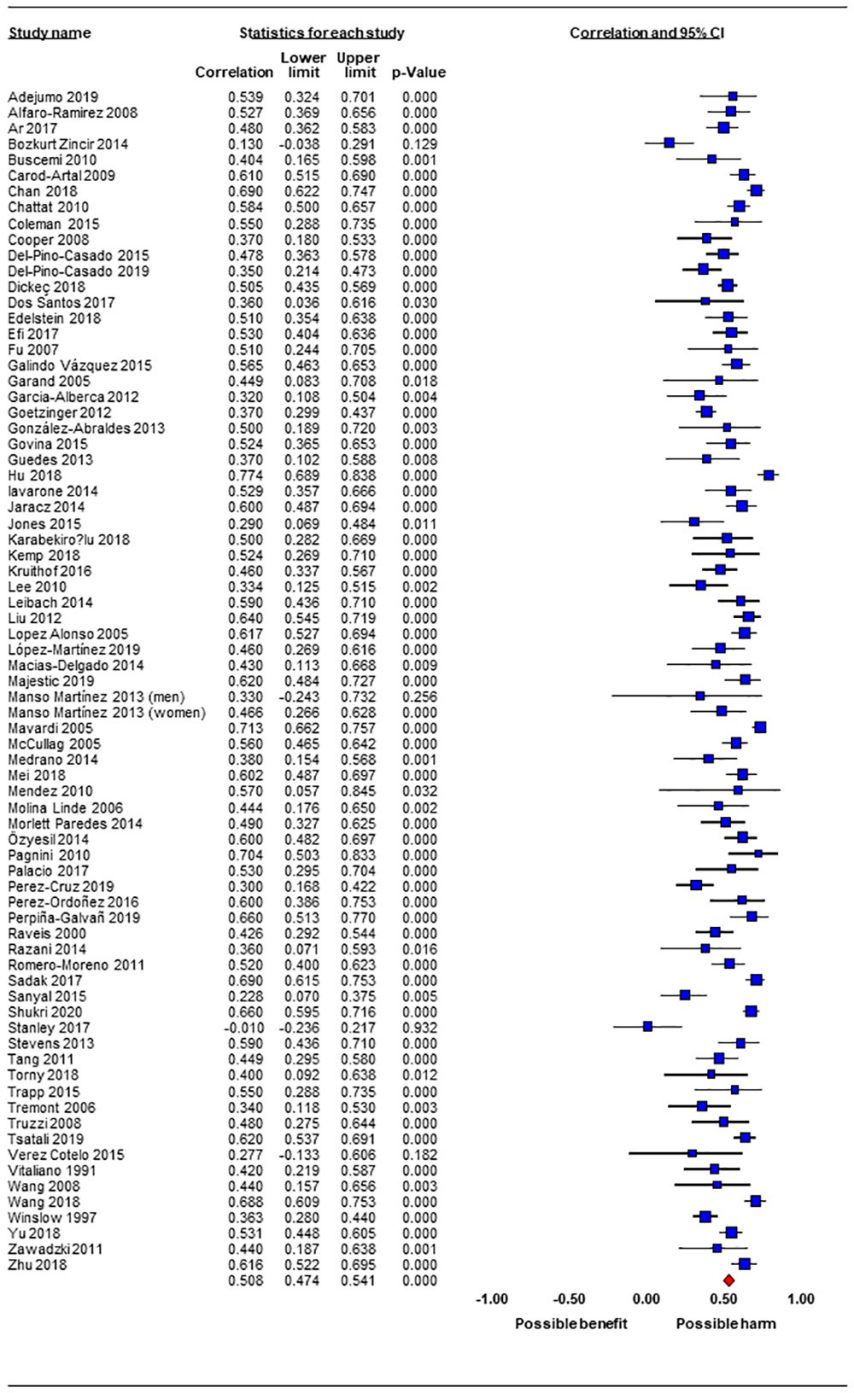
Forest plot of the results of the meta-analysis.

Results of the meta-analyses showed that the combined effect of the association between subjective caregiver burden and anxiety symptoms was r = 0.51 (95% confidence interval [CI] = 0.47; 0.54; 75 samples; Table 3), indicative of a large positive association. Fig 2 presents the results of the forest plot showing the effect of each individual study. All studies except one [96] reported positive correlations and all studies except four [47,77,96,104] reported a statistically significant association between subjective caregiver burden and anxiety symptoms. The total number of participants included in the meta-analysis was n = 10,122 and n = 135 was the mean number of participants per study. There was no heterogeneity amongst individual studies (Q = 63.47, degrees of freedom = 74, p = 0.80, I^2^ = 0.0%).

#### Quality ratings

Table 2 shows quality ratings of included studies. Most used non-probabilistic samples (70 studies with 71 samples), whereas all met criterion 2 reporting on a reliable and valid measure of subjective caregiver burden and anxiety symptoms. Only 20 studies (26.7%) reported controlling for several confounders which included function, behavioral and psychological symptoms, cognitive impairment, and intensity of care provided [17,48,53,60,71,74,77,86,88,91,92,100–104,106,107,111,112].

**Table 2:** Quality assessment of the studies included in the review (I).

Studies	Probabilistic sampling	Reliability and validity of measures	Control of confounders	Absence of attrition
Adejumo et al., 2019 [44]	-	+	?	N/A
Alfaro-Ramirez del Castillo et al., 2008 [45]	-	+	?	N/A
Ar, 2017 [46]	-	+	?	N/A
Bozkurt Zincir et al., 2014 [47]	-	+	?	N/A
Buscemi et al., 2010 [14]	-	+	-	N/A
Carod-Artal et al., 2009 [48]	-	+	+	N/A
Chan et al., 2018 [49]	-	+	?	N/A
Chattat et al., 2011 [50]	-	+	?	N/A
Coleman et al., 2015 [51]	-	+	?	N/A
Cooper et al. 2008 [52]	-	+	?	-
del-Pino-Casado et al., 2015 [53]	+	+	+	N/A
del-Pino-Casado et al., 2019 [54]	+	+	+	+
Dikeç et al., 2018 [55]	-	+	?	N/A
Dos Santos et al., 2017 [56]	-	+	?	N/A
Edelstein et al., 2018 [15]	-	+	?	N/A
Efi et al., 2017 [57]	-	+	?	N/A
Fu et al., 2007 [58]	-	+	?	N/A
Galindo Vázquez et al., 2015 [59]	-	+	?	N/A
Garand et al., 2005 [60]	-	+	+	N/A
Garcia-Alberca et al., 2012 [11]	-	+	?	N/A
Goetzinger et al., 2012 [61]	-	+	-	N/A
Gonzalez-Abraldes et al., 2013 [62]	-	+	?	N/A
Govina et al., 2015 [63]	-	+	?	N/A
Guedes and Pereira, 2013 [64]	-	+	?	N/A
Hu et al., 2018 [13]	-	+	?	N/A
Iavarone et al., 2014 [65]	-	+	?	N/A
Jaracz et al., 2014 [66]	-	+	?	N/A
Jones et al., 2015 [67]	-	+	?	N/A
Karabekiroglu et al., 2018 [68]	-	+	?	N/A
Kemp et al., 2018 [69]	-	+	?	N/A
Kruithof et al., 2016 [12]	-	+	?	+
Lee et al., 2010 [70]	-	+	?	N/A
Leibach et al., 2014 [71]	-	+	+	N/A
Liu et al., 2012 Liu et al. [72]	-	+	?	N/A
López Alonso and Moral Serrano, 2005 [73]	+	+	?	N/A
López-Martínez, 2019 [74]	+	+	+	-
Macías-Delgado Yanet et al., 2014 [75]	-	+	?	N/A
Majestic and Eddington, 2019 [76]	-	+	?	N/A
Manso Martínez et al., 2013 (men) [77]	-	+	-	N/A
Manso Martínez et al., 2013 (women) [77]	-	+	+	N/A
Mavardi et al., 2005 [78]	-	+	?	N/A
McCullagh et al., 2005 [79]	-	+	?	-
Medrano et al., 2014 [80]	-	+	?	N/A
Mei et al., 2018 [81]	-	+	?	N/A
Méndez et al., 2010 [82]	-	+	?	N/A
Molina Linde and Iañez Velasco 2006 [83]	-	+	-	N/A
Morlett Paredes, 2014 [84]	-	+	?	N/A
Özyesil et al., 2014 [85]	-	+	?	N/A
Pagnini et al., 2010 [86]	-	+	+	N/A
Palacio et al., 2018 [87]	-	+	?	N/A
Perez Cruz et al., 2019 [88]	-	+	+	N/A
Pérez-Ordóñez et al., 2016 [17]	-	+	+	N/A
Perpiña-Galvañ et al., 2019 [89]	-	+	?	N/A
Raveis et al., 2000 [90]	-	+	-	N/A
Razani et al., 2014 [91]	-	+	+	?
Romero-Moreno et al., 2011 [92]	-	+	+	N/A
Sadak et al., 2017 [93]	-	+	?	N/A
Sanyal et al., 2015 [94]	-	+	?	N/A
Shukri et al., 2020 [95]	-	+	?	N/A
Stanley et al., 2017 [96]	-	+	?	N/A
Stevens et al., 2013 [111]	-	+	+	N/A
Tang et al., 2011 [98]	-	+	?	N/A
Torny et al., 2018 [99]	-	+	?	N/A
Trapp et al., 2015 [100]	-	+	+	N/A
Tremont et al., 2006 [101]	-	+	+	N/A
Truzzi et al., 2008 [102]	-	+	+	N/A
Tsatali et al., 2019 [103]	-	+	+	N/A
Vérez Cotelo et al., 2015 [104]	-	+	+	N/A
Vitaliano et al., 1991 [105]	-	+	?	+
Wang et al., 2008 [106]	-	+	?	N/A
Wang et al., 2018 [16]	-	+	+	N/A
Winslow, 1997 [107]	-	+	+	+
Yu et al., 2018 [108]	-	+	?	N/A
Zawadzki et al., 2011 [109]	-	+	?	N/A
Zhu and Jiang, 2018 [110]	-	+	?	+

Notes:

^(+)^ characteristic is present;

^(−)^ characteristic is absent;

^(?)^ there is not enough information to assess bias.

#### Publication bias and sensitivity analyses

**Fig 3:**
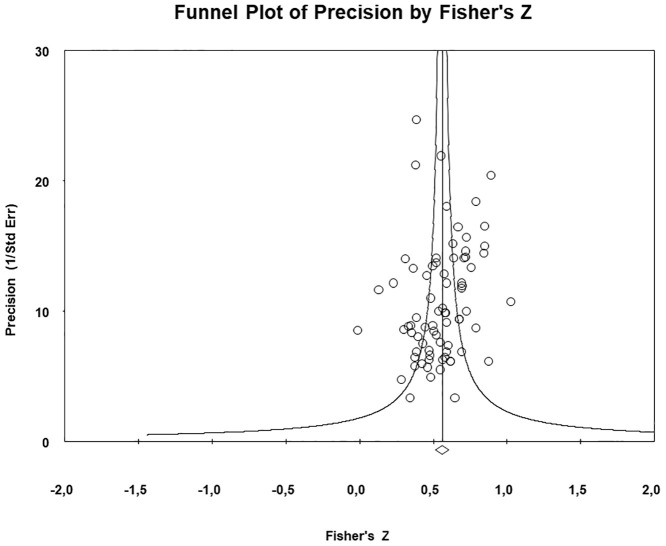
Funnel plot of the results of the meta-analysis.

Inspection of the funnel plot (see Fig 3) indicates asymmetry, even though there are no extreme values. Results of the Egger test showed that there is a low risk of publication bias overall (p = 0.13). We calculated additional analyses to investigate publication bias based on the Hedges and Pigott method [113] in order to calculate the power of the Egger test, obtaining 99%. The combined effect value using the Trim and Fill method [35] (r = 0.57) varied by 11.8% with respect to initial calculations (0.57), indicative of a small effect of publication bias. Sensitivity analyses eliminating one study at a time showed that the combined r-value differed less than 1.8% from the original combined effect (from 0.50 to 0.51), demonstrating the robustness of the findings.

#### Subgroup analyses

We performed several subgroup analyses to investigate the effect of several moderators in the association between subjective caregiver burden and anxiety symptoms. As can be seen from Table 3, there were no differences between cross-sectional (r = 0.52; IC 95% = 0.48, 0.55; 67 samples) and longitudinal studies (r = 0.43; 95% CI = 0.36, 0.49; 8 samples), in terms of the estimate of the effect. We found no differences between studies using probabilistic samples (r = 0.49; 95% CI = 0.35, 0.60; 4 samples) versus those that did not (r = 0.51; 95% CI = 0.47, 0.54; 71 samples). Similarly, the effect estimate did not change between studies that controlled for several confounders (r = 0.50; 95% CI = 0.44, 0.56; 20 samples) versus those that did not (r = 0.51; 95% CI = 0.47, 0.55; 55 samples). We further investigated whether care recipient characteristics moderated the effect. As shown in Table 3 there were no differences between studies reporting on associations in carers of people with dementia, carers of frail older people, and carers of cancer or stroke survivors.

**Table 3:** Summary of findings of the different meta-analyses.

	k	N	N/k	r	95% CI		Heterogeneity		Inconsistency	One study	Publication bias			
					Lower limit	Upper limit	Q (df)	p	I^2^	removed (% var)	Funnel plot	p for Egger test	Trim & Fill	
													Estimate	% var
All samples	75	10,122	135.0	0.51	0.47	0.54	63.47 (74)	0.80	0.0	1.8	Asymmetric	0.13	0.57	11.8
Study design														
Cross-sectional	67	8,780	131.0	0.52	0.48	0.55	57.80 (66)	0.75	0.0	1.4	Asymmetric	0.06	0.58	11.5
Longitudinal	8	1,342	167.8	0.43	0.36	0.49	5.7 (7)	0.58	0.0	8.5	Asymmetric	0.89	0.43	0.0
Sampling														
Probabilistic	4	674	168.5	0.49	0.35	0.60	2.59 (3)	0.46	0.0	12.0	Asymmetric	0.7	0.53	8.2
Non probabilistic	71	9,448	133.1	0.51	0.47	0.54	59.80 (70)	0.80	0.0	1.4	Asymmetric	0.14	0.58	13.7
Confounders														
Control	20	2,554	127.7	0.50	0.44	0.56	15.48 (19)	0.69	0.0	3.0	Symmetric	0.69	0.51	2.0
No control	55	7,568	137.6	0.51	0.47	0.55	47.55 (54)	0.72	0.0	1.8	Asymmetric	0.06	0.58	13.7
Care recipients														
Dementia	24	3,055	127.3	0.50	0.43	0.56	14.94 (23)	0.90	0.0	2.4	Asymmetric	0.06	0.57	13.8
Frail older people	12	1,632	136.0	0.51	0.42	0.59	8.40 (11)	0.68	0.0	4.3	Asymmetric	0.34	0.53	3.9
Cancer	12	1,083	90.3	0.52	0.46	0.57	10.87 (11)	0.45	0.0	2.9	Asymmetric	0.95	0.49	6.0
Stroke	8	1,353	169.1	0.58	0.51	0.65	8.34 (7)	0.30	16.0	5.0	Symmetric	0.58	0.60	3.4

Abbreviations: K = number of samples; N = number of participants in each meta-analysis; N/k = mean of participants per study; r = correlation coefficient; CI = confidence interval; Q = Cochran Q; df = degrees of freedom; p = p-value; I^2^ = degree of inconsistency; % var = percentage of variation respect to the original effect estimate (r).

## Discussion

Our study is the first systematic review and meta-analysis of the worldwide literature providing an estimate of the magnitude of the association between subjective caregiver burden and anxiety symptoms in informal carers. Results of our meta-analyses found a large positive association between subjective caregiver burden and anxiety symptoms as predicted by theory [10] and empirical work in the area [11–13]. The results showed robustness to the effects of several modifiers such as study design (cross-sectional vs. longitudinal), sampling methods used (probabilistic or non-probabilistic), control of confounders, and care-recipient characteristics. An important strength of this study was quantifying the relationship between subjective caregiver burden and anxiety in informal carers across all caregiving groups. Our findings that the estimate of the association was similar across different groups of caregivers supports the wider applicability of our findings.

Our review provides important new knowledge in the area by rigorously evaluating the methodological quality of evidence to date and assessing the influence of important confounders such as objective burden and the potential effect of publication bias. Our findings have high consistency and precision overall, given that the risk of publication bias was small, and sensitivity analyses showed no substantial variation in the combined effect. There were no differences in the combined effect between cross-sectional and longitudinal studies providing further support for the robustness and precision of our results. However, the observed differences in the point estimates and the slight overlap of the confidence intervals indicate a possible overestimation of the effect in cross-sectional studies.

As predicted by individual studies subjective caregiver burden was associated with high levels of clinically significant anxiety in carers [11–13]. The strength of the association observed in our meta-analysis suggests that subjective caregiver burden is an important determinant of anxiety-related psychological distress in carers. This finding indicates that alleviating subjective caregiver burden will protect carers from experiencing high levels of anxiety symptoms. Therefore, interventions aimed at early detection and prevention of subjective caregiver burden and anxiety symptoms such as psychoeducation and skills building interventions and access to respite care should be routinely offered to family carers [114,115]. Interestingly our analyses indicated that this association was not influenced by objective burden parameters such as levels of every-day function, behavioural and psychological symptoms or presence of cognitive impairment in the care recipient.

### Implications for clinical practice and research

Within the caregiving context, our finding that carers reporting high levels of subjective caregiver burden are more likely to experience clinically significant anxiety is important. Anxiety is a highly distressing condition, increasing risk of cardiovascular disease, compromising carers’ physical health, and lowering their quality of life [116]. Screening for, and addressing high caregiver burden therefore may improve psychiatric and physical health outcomes for carers [112]. More widely, interventions that prevent caregiver burden supporting carers to cope with the demands of caregiving duties such as access to day care services [115] and psychotherapeutic support [114] would be an important investment for clinical services worldwide. Future research however is needed to explore some of the issues raised by our meta-analysis. For example, future studies are required to identify other individual and environmental factors that may moderate the association between subjective caregiver burden and anxiety.

Furthermore, research has shown that spousal and adult-child caregivers differ on several dimensions of subjective caregiver burden [117], and on the objective stressors that influence levels of burden experienced [118]. For example, behavioral and psychological symptoms are more strongly associated with subjective burden in spousal as opposed to adult-child caregivers [27]. A better understanding of these associations and how interventions can better address the needs of spousal versus adult-child caregivers, will increase their effectiveness, and should be a focus of future work in the area.

Prospective studies investigating prognosis and long-term outcomes for carers experiencing high levels of anxiety and subjective caregiver burden are currently lacking. Further longitudinal studies are required that evaluate how subjective caregiver burden and anxiety symptoms may change over time.

### Limitations

Despite the rigour of our meta-analysis the results of our review should be interpreted with caution. First, the design of the longitudinal studies included in our review does not allow us to conclude whether reverse causation between subjective caregiver burden and anxiety symptoms is present or absent. An important potential bias could be that higher levels of subjective caregiver burden reported by carers may not be burden attributed to the caregiving experience per se but may instead reflect high levels of anxiety symptoms experienced by carers. Second, we were not able to control for confounders other than objective caregiver burden, such as stressful life events, social support, coping or socio-economic factors that are related to anxiety symptoms [18] and our meta-analysis was not registered online. Moreover, some of the studies included in our meta-analysis did not primarily investigate the association between subjective caregiver burden and anxiety symptoms, and this may have influenced the accuracy of the effect observed in our meta-analyses. Lastly, we were not able to examine how being a spousal versus an adult-child caregiver influences the association between subjective caregiver burden and anxiety and the differential impact of moderators of this association amongst spousal versus adult-child caregivers.

## Conclusion

Despite limitations, this is an important study providing the first quantitative estimate of the association of subjective caregiver burden and anxiety symptoms in informal caregivers. This meta-analysis suggests that subjective caregiver burden has a large association with anxiety symptoms, and this relationship is found across all caregiving groups. It does not appear to be affected by study design (cross-sectional vs. longitudinal), sampling, control for confounders or care-recipient characteristics. Results provide support for a newly focus on interventions to detect subjective caregiver burden and prevent clinically significant anxiety symptoms for the increasing number of informal caregivers worldwide.

## Supporting information

S1 Checklist PRISMA checklist. (DOC)

S1 Appendix Abbreviations of measures. (DOCX)
