# Supplementary material for: Subjective caregiver burden and anxiety in informal caregivers: A systematic review and meta-analysis
Source: PLoS One. 2021 Mar 1;16(3):e0247143. doi: 10.1371/journal.pone.0247143 (PMC7920375; doi:10.1371/journal.pone.0247143)
Supplement: S1 Appendix — (DOCX) [file pone.0247143.s002.docx]

**S1 APPENDIX: ABBREVIATIONS OF MEASURES**

**SUBJECTIVE BURDEN**

BASC Brief Assessment Scale for Caregivers of the Medically Ill

BCOS Bakas Caregiving Outcomes Scale

BSFC Burden Scale for Family Caregivers

CBI Caregiver Burden Inventory

CBS 1 Caregiver Burden Scale (Andren & Elmstahl)

CBS 2 Caregiver Burden Scale (Gerritsen & Van der Ende)

CSI Caregiver Strain Index

KCSS Kingston Caregiver Stress Scale

OCBS Oberst Caregiving Burden Scale

PFBS Perceived Family Burden Scale

SBS Subjective Burden Scale

SCB Scale for Caregiver Burden

SCB Screen of Caregiver Burden

ZBI Zarit Burden Interview

**ANXIETY**

BAI Beck Anxiety Inventory

BSI Brief Symptom Inventory

CRA Caregiving Reaction Assessment

DASS Depression Anxiety Sttress Scale

GAD Generalized Anxiety Disorder Assessment

HADS Hospital Anxiety Depression Scale

HARS Hamilton Anxiety Rating Scale

HSCL Hopkins Symptom Checklist

ISRA Inventario de situaciones y respuestas de ansiedad [Anxiety Situations and Responses Inventory]

MLSI Multimodal Life Story Inventory

POMS Profile of Mood States

SAS Self-Rating Anxiety Scale

SCL Symptoms Check-List

STAI State Trait Anxiety Inventory
